# MRI radiomics predicting cribriform growth informs active surveillance decision in intermediate-risk prostate cancer

**DOI:** 10.1186/s41747-026-00728-9

**Published:** 2026-05-13

**Authors:** Mar Fernandez Salamanca, Rita Simões, Malgorzata Deręgowska-Cylke, Eduardo Pais Pooch, Pim J. van Leeuwen, Henk G. van der Poel, Marcos A. S. Guimaraes, Uulke A. van der Heide, Ivo G. Schoots

**Affiliations:** 1 Department of Radiology, The Netherlands Cancer Institute, Plesmanlaan 121, 1066CX Amsterdam, The Netherlands; 2 Department of Radiation Oncology, The Netherlands Cancer Institute, Plesmanlaan 121, 1066CX Amsterdam, The Netherlands; 3 Department of Radiology, Medical University of Warsaw, Żwirki i Wigury 61, 02-091 Warsaw, Poland; 4 GROW Research Institute for Oncology and Reproduction, Maastricht University, Universiteitssingel 40, 6229ER Maastricht, The Netherlands; 5 Department of Urology, The Netherlands Cancer Institute, Plesmanlaan 121, 1066CX Amsterdam, The Netherlands; 6 Department of Urology, Amsterdam University Medical Centers, Meibergdreef 9, 1105AZ Amsterdam, The Netherlands; 7 Department of Pathology, The Netherlands Cancer Institute, Plesmanlaan 121, 1066CX Amsterdam, The Netherlands; 8 Department of Radiology & Nuclear Medicine, Erasmus University Medical Center, Dr. Molewaterplein 40, 3015GD Rotterdam, The Netherlands

**Keywords:** Active surveillance, Cribriform growth, Magnetic resonance imaging, Prostate cancer, Radiomics

## Abstract

**Objective:**

Accurate exclusion of cribriform growth (GP4Crib+), an adverse histologic feature in prostate cancer, remains a challenge in selecting intermediate-risk patients for active surveillance (AS). This study evaluates whether an MRI-based radiomics model predicting GP4Crib+ can support AS inclusion decisions under the assumption that intermediate-risk men without GP4Crib+ could be safely managed with AS.

**Materials and methods:**

This single-center retrospective study was approved by the institutional review board (IRBd21-108) and included men with Cambridge Prognostic Group (CPG) -1, CPG-2, or CPG-3 (Gleason grade (GG) 2) prostate cancer who underwent MRI and radical prostatectomy. In this cohort, a previously trained radiomics model was applied across the whole prostate to generate voxel-wise GP4Crib+ probability maps, to ultimately identify men with GP4Crib. This model was tested in two scenarios: (1) CPG-1+2, and (2) CPG-1+2+3(GG2) patients on biopsy. The reference scenario was CPG-1 men to AS (guideline recommendations).

**Results:**

We included 127 patients (median age 66 years, interquartile range 47‒78). Standalone radiomics performance was moderate (area under the receiver operating characteristic curve (AUROC): 0.68 overall; 0.60 for CPG-2; and 0.70 for CPG-3(GG2)). At a 0.60 probability threshold, the model reduced overtreatment by 9% in CPG-1 + 2 men (with 1% increase in undertreatment). In CPG-1+2+3(GG2) men, the model reduced overtreatment by 8%, maintaining low undertreatment (3%). Most false negatives involved smaller than 1.5-mm cribriform foci.

**Conclusion:**

Our MRI-based GP4Crib+ radiomics model is able to support AS decisions in men with CPG-1+2+3(GG2). Despite modest standalone diagnostic performance, its integration into the clinical work-up may help safely expand AS selection to intermediate-risk men, ultimately reducing overtreatment.

**Relevance statement:**

MRI-based radiomics may support noninvasive exclusion of cribriform growth to guide active surveillance eligibility in intermediate-risk prostate cancer, enabling safer management decisions and reducing unnecessary treatment.

**Key Points:**

- Prostate cancer cribriform growth is difficult to detect but crucial for treatment decisions.
- MRI radiomics helps exclude cribriform growth in intermediate-risk prostate cancer patients.
- Radiomics-informed decisions reduced overtreatment under structured AS scenarios.

**Graphical Abstract:**

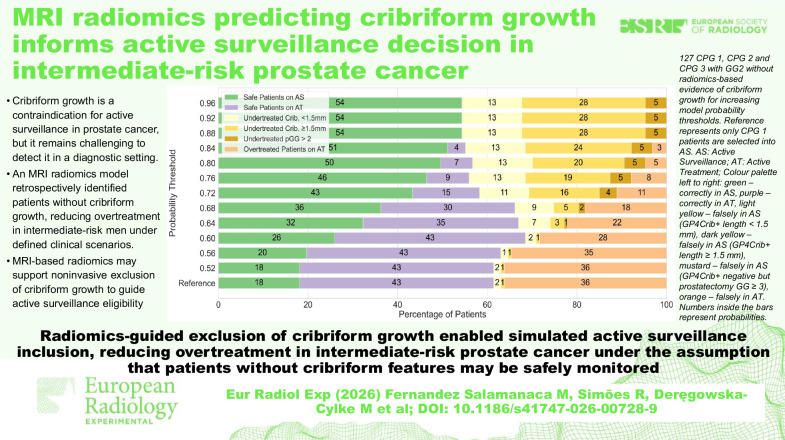

## Background

Magnetic resonance imaging (MRI) has become central to the diagnosis, risk stratification, and management of prostate cancer. Its capacity to noninvasively characterize tumor biology, to guide targeted biopsy, and to inform treatment decisions has significantly transformed clinical workflows [1, 2]. Among its growing clinical applications, MRI is increasingly being explored for its potential to detect histologically aggressive features such as cribriform growth, particularly in patients with intermediate-risk disease [3–5].

Cribriform growth, one of the basic patterns of Gleason pattern (GP) 4 (GP4Crib+), is strongly associated with worse clinical outcomes, including higher rates of biochemical recurrence, metastasis, and prostate cancer-specific mortality [6–9]. Absence of GP4Crib+ in men with Gleason grade (GG) 2 and GG3 prostate cancer shows a similar risk to men with GG1 and GG2 disease, respectively [10]. Within the clinical context of safely expanding inclusion criteria for active surveillance (AS), men without GP4Crib+ could therefore potentially opt for AS.

The current prostate cancer risk grouping excludes imaging information and is only based on clinical parameters and biopsy results [11–13]. According to these guidelines, AS is primarily recommended for low-risk patients (Cambridge Prognostic Group (CPG) 1), with the addition of selected CPG 2 patients considered only after multidisciplinary discussion. However, real-world clinical practice is gradually evolving toward broader inclusion of CPG 2 patients into AS protocols, particularly in settings where MRI and targeted biopsy improve risk assessment and tumor characterization. Yet, GP4Crib+, as a key prognostic driver, is not explicitly considered in current risk grouping and thus is not routinely used in treatment guidance.

The detection of GP4Crib+ at biopsy remains challenging, particularly in patients with GG2 disease, due to sampling errors, tumor heterogeneity, and reader variability [14, 15]. This highlights the need for noninvasive imaging biomarkers that can assist in detecting or excluding GP4Crib+.

This study investigates whether a previously developed radiomics model—trained to distinguish GP4Crib+ from other GPs (GP3 and GP4Crib-) based on apparent diffusion coefficient (ADC) features [16]—can inform patient-level decision-making when applied across the entire prostate gland. The focus of this work is not only on evaluating the model’s performance at the patient level, but also on understanding how excluding radiomics-predicted cribriform growth could impact AS eligibility decisions under the assumption that men with CPG-2 or CPG-3(GG2) and no GP4Crib+ may be suitable for AS.

## Methods

### Study population

**Fig. 1:**
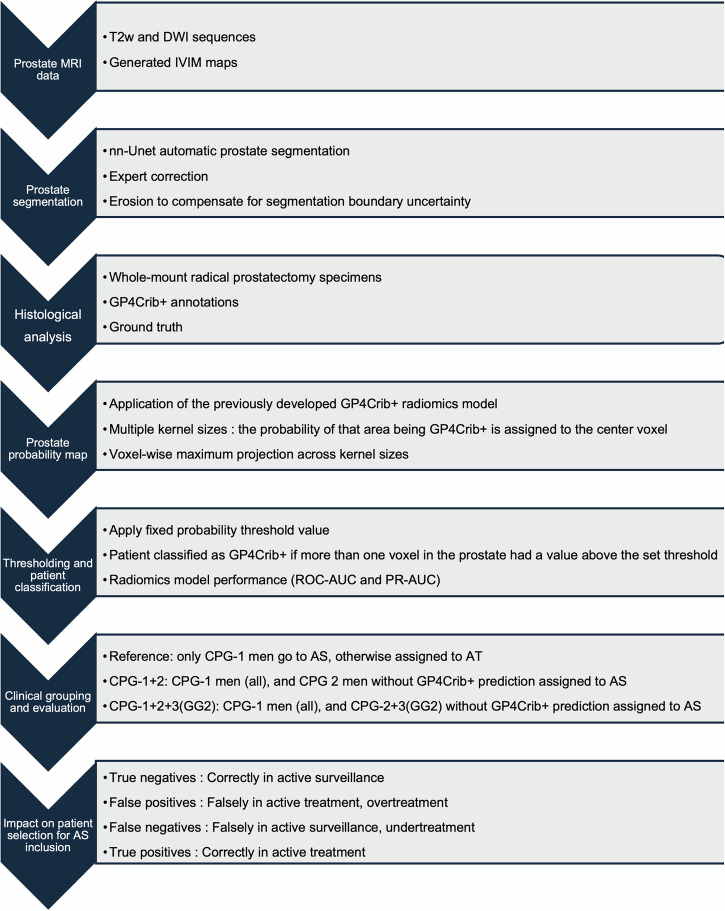
Project pipeline. AS, Active surveillance; DWI, Diffusion-weighted imaging; GP4Crib+, Cribriform growth; IVIM, Intravoxel incoherent motion; T2w, T2-weighted

The study was approved by the institutional review board (IRBd21-108), and informed consent was waived due to the retrospective nature of the analysis. This retrospective study included men with biopsy-proven prostate cancer who underwent radical prostatectomy at the Netherlands Cancer Institute between January 2010 and December 2020. Exclusion criteria included prior transurethral resection of the prostate, neoadjuvant systemic therapy or radiotherapy, or incomplete/poor-quality MRI or pathology specimens. Inclusion criteria required patients to have undergone preoperative MRI, biopsy-confirmed cancer, and to have undergone a complete radical prostatectomy with Hematoxylin and Eosin staining. For this study, only patients with CPG-1, CPG-2, and CPG-3 (GG2) were used. The clinical information of the final 127 patients can be seen in Table 1. The successive steps in the method section towards outcome measurements are illustrated in Fig. 1.

**Table 1:** Patient demographics for the full cohort and the different clinical groups

Finding	CPG-1	CPG-1+2	CPG-1+2+3(GG2)
Patients	*n* = 26	*n* = 98	*n* = 127
Age (years)	63 (47–74)	66 (47–78)	66 (47–78)
PSA (ng/mL)	6.7 (2.8–9.2)	7.1 (2.8–18.7)	7.7 (2.8–19)
Biopsy GG			
1	26 (100%)	33 (34%)	33 (26%)
2	–	65 (66%)	94 (74%)
3	–	–	–
4	–	–	–
5	–	–	–
Clinical T-stage			
1	10 (38%)	30 (31%)	37 (29%)
2	16 (62%)	68 (69%)	90 (71%)
3	–	–	–
Missing	–	–	–
Cambridge prognostic group			
1	26 (100%)	26 (27%)	26 (20%)
2	–	72 (73%)	72 (57%)
3	–	–	(with GG2) 29 (23%)
4	–	–	–
5	–	–	–
Incomplete	–	–	–
Prostatectomy GG			
1	16 (62%)	23 (24%)	23 (18%)
2	10 (38%)	63 (64%)	82 (65%)
3	–	9 (9%)	17 (13%)
4	–	2 (2%)	3 (2%)
5	–	1 (1%)	2 (2%)
Prostatectomy T-stage			
2	21 (81%)	73 (74%)	91 (72%)
3 or higher	5 (19%)	25 (26%)	36 (28%)
Cribriform growth presence			
No	23 (88%)	62 (63%)	75 (59%)
Yes	3 (12%)	36 (37%)	52 (41%)

Data are medians (min–max) or absolute frequencies (percentages)

*CPG* Cambridge prognostic group, *GG* Grade group, *PSA* Prostate-specific antigen

### MRI acquisition and maps generation

MRI scans were performed using 1.5-T (Achieva (*n* = 2)) and 3-T systems (Achieva (*n* = 46), Achieva dStream (*n* = 63), Ingenia (*n* = 16), Philips Healthcare, Best, the Netherlands). The imaging protocol included axial turbo spin-echo T2-weighted sequences with repetition times ranging from 2,175 to 8,869 ms and echo times from 110 to 130 ms. Field of view dimensions ranged from 140 × 140 mm² to 282 × 282 mm², with slice thickness set between 2.5 and 4 mm. In addition, diffusion-weighted imaging (DWI) was acquired using two-dimensional single-shot echo planar imaging techniques. These DWI sequences employed fields of view of 180 × 180 mm² to 381 × 381 mm², with slice thickness between 2.73 and 4 mm and in-plane spatial resolution varying from 0.56 × 0.56 mm² to 1.2 × 1.2 mm². The range of *b*-values used for diffusion encoding included combinations (0, 200, 800 s/mm² and 0, 50, 300, 800 s/mm²), with some cases incorporating an additional high *b*-value (1,400–2,000 s/mm²). ADC and fractional blood volume maps were generated using an intravoxel incoherent motion (IVIM) model, implemented via a segmented fitting technique^1^, as described previously [17].

### Prostate segmentation

Prostates were automatically segmented on T2-weighted images using a nnU-Net algorithm trained on 772 T2-weighted scans of 650 patients from the Prostate-MRI-US-Biopsy dataset [18, 19] and manually checked by two independent observers (M.F.S. and M.D.C., each with 1–2 years of prostate MRI reading experience). Prostate segmentations were coregistered to ADC maps, when needed, using the 3DSlicer Transform tool [http://www.slicer.org/]. To mitigate segmentation uncertainty, prostate masks on the ADC maps were eroded by removing a single boundary voxel using the binary erosion function from the SciPy library (version 1.11.1). The erosion was performed by using a cylindrical structuring element in the *xy* plane of the ADC maps. Erosion was restricted to slices where prostate segmentation was also present in the adjacent upper and lower slices, to avoid edge-related uncertainties.

### Histological analysis

Whole-mount radical prostatectomy specimens were formalin-fixed, sectioned at 3-mm intervals, and histologically analyzed by a dedicated uropathologist (M.A.S.G, 10 years’ experience) using SlideScore software [https://www.slidescore.com/] and a 40× magnification. Each cancerous lesion was graded according to the 2019 International Society of Urological Pathology recommendations, with additional annotations of GP3, GP4Crib+, and GP4Crib- regions. Patients were classified as GP4Crib+ positive at the patient level if at least one GP4Crib+ region was identified anywhere in the prostate specimen, serving as the ground truth.

### Prostate probability map: application of the GP4Crib+ radiomics model to the whole prostate

A previously developed logistic regression radiomics model differentiated GP4Crib+ from other GPs (*i.e.*, GP3 and GP4Crib −) using imaging features extracted from histologically proven GP areas [16]. The model was trained on 465 histology-proven GP areas from 291 men who underwent radical prostatectomy, with manual co-registration of whole-mount histopathology to MRI. To minimize partial volume effects and registration inaccuracies, isotropic erosion was applied to the annotated areas prior to feature extraction. Radiomics features were extracted from T2-weighted imaging, ADC maps, and fBV maps. Feature selection was performed using the minimum redundancy maximum relevance algorithm, and the optimal logistic regression model used only one feature: the 90th percentile of ADC values. This feature, negatively associated with cribriform growth, showed the highest discriminatory power for GP4Crib+. This feature was negatively associated with cribriform growth, consistent with the dense cellular architecture typically found in such regions.

In the current study, we build on these findings by applying that feature-driven model across the entire prostate gland in a voxel-wise manner. Rather than characterizing known regions, our aim was to use these imaging characteristics to detect which patients may have GP4Crib+ presence—enabling a patient-level assessment to inform AS eligibility.

Using a sliding-window approach, the model was applied voxel-wise to ADC maps, generating a spatial probability map for each patient that reflects the likelihood of cribriform growth throughout the prostate. In this process, square-shaped windows of varying sizes—specifically, 5 × 5, 7 × 7, 9 × 9, 11 × 11, and 13 × 13 voxels—were systematically applied to the ADC images based on their respective in-plane resolution and slice thickness. Within each window, the 90th percentile ADC value was calculated, and this feature was used as input to the logistic regression model. The model output was a probability score indicating the likelihood that the middle voxel within that window was GP4Crib+.

To account for the overlap between sliding windows during model inference, a probability aggregation step was introduced. Each voxel in the prostate could be included in multiple windows, each producing a potentially different GP4Crib+ probability. To consolidate these predictions, the maximum probability value across all windows covering a specific voxel was selected. This “maximum projection” approach ensured that the most suspicious signal was retained for each voxel, resulting in a final GP4Crib+ probability map spanning the entire prostate.

### Thresholding and patient classification: radiomics model performance

Each patient has a voxel-level probability map representing the predicted probability of cribriform presence across the prostate. A continuous patient-level score was defined as the maximum probability value across all voxels within the prostate mask. This maximum probability represents the model’s highest confidence of cribriform presence for that patient. The model performance was evaluated using the receiving operation curve and precision-recall area under the curve analysis (ROC-AUC and PR-AUC, respectively), and 95% confidence intervals (CIs) were assessed using bootstrapping with *n *= 1,000. All analyses were conducted using Python v3.11 (Python Software Foundation, https://www.python.org/).

### Clinical grouping and evaluation

Following a standalone evaluation of the radiomics model’s predictive performance, we then assessed its clinical utility by simulating two AS inclusions under the assumption that selected CPG-2 and CPG-3(GG2) patients without cribriform growth and GG < 3 could be managed with AS. For this clinical simulation, binary classification was performed by applying probability thresholds to the maximum probability value per patient. A patient was classified as radiomics-predicted cribriform-positive if their maximum voxel probability exceeded the chosen threshold, and cribriform-negative otherwise. Multiple probability thresholds were evaluated to assess the tradeoff between sensitivity and specificity in the context of treatment classification.

The first AS inclusion scenario includes patients classified as CPG-1 and CPG-2 disease (CPG-1+2), an approach increasingly adopted in clinical practice. Under this scenario, patients in the CPG-1 group are eligible for AS, in line with current practice. The radiomics model was applied to the CPG-2 subgroup. Patients would have been recommended for AS only if the model predicted the absence of cribriform growth; otherwise, they would have been guided to AT.

The second AS inclusion scenario expands eligibility to also include CPG-3 patients with only GG2 disease (CPG-1+2+3(GG2)). Again, the radiomics model was applied to the CPG-2 and CPG-3(GG2) subgroups, with AS eligibility dependent on a negative prediction for cribriform growth.

In both scenarios, all CPG-1 patients were automatically assigned to AS without model application, serving as a clinical benchmark [12, 13]. For comparison, we defined the reference scenario, where the radiomics model was not applied, as only CPG-1 patients were considered eligible for AS.

### Impact on patient selection for AS inclusion

This study investigates how excluding cribriform growth using an MRI-based radiomics model might influence patient selection for AS, under the assumption that CPG-2 or CPG-3(GG2) patients who lack cribriform growth on final prostatectomy could be safely managed by AS. Therefore, we use the GP4Crib+ predictions of the radiomics model in men categorized as CPG-2 or CPG-3(GG2) to define the appropriateness of the proposed management scenarios (*i.e.*, either AS or AT) based on the histological ground truth. No patients in this cohort were managed with AS; the analysis is retrospective and simulates hypothetical treatment allocation.

GP4Crib+ patients at prostatectomy, who were correctly classified by the model (true positives), would have been appropriately managed by active treatment/AT, suggesting no undertreatment (purple color).

GP4Crib- men with GG < 3 cancer at prostatectomy, who were correctly classified as GP4Crib- by the model (true negatives), would have been appropriately managed by AS (green color), suggesting a reduction in active treatment (*i.e.*, overtreatment).

GP4Crib+ men at prostatectomy, who were incorrectly classified by the model as CP4Crib- (false negatives), would have been inappropriately managed by AS, and therefore would have been undertreated. Due to voxel size limitations, MRI is expected to determine GP4Crib+ cancer above 1.5 mm (intermediate yellow), while not below this diameter (light yellow). GP4Crib- patients with GG ≥ 3 cancer, who were correctly classified as GP4Crib- by the model, however not eligible for AS due to GG ≥ 3 cancer (false negative), would have also been considered to be inappropriately managed and therefore undertreated (dark yellow).

GP4Crib- patients with GG < 3 cancer on prostatectomy, who were incorrectly classified as GP4Crib+ by the model (false positives), would have been inappropriately managed by active treatment/AT; these men would have been overtreated (orange color).

This 2 × 2 contingency framework (Table 2) allowed for quantitative analysis of the model’s impact on both overtreatment (false positives) and undertreatment risks (false negatives).

**Table 2:**
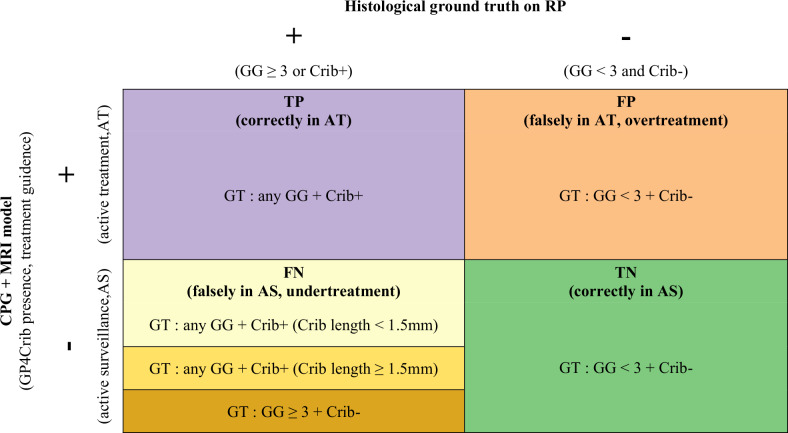
Patient-level treatment classification in radiomics-informed clinical decision strategies compared to histological ground truth from radical prostatectomy

*CPG* Cambridge prognostic group, *Crib* Cribriform growth, *FN* False negative, *FP* False positive, *GG* Grade group, *GT* Ground truth, *RP* Radical prostatectomy, *TN* True negative, *TP* True positive

## Results

### Patients

Patient demographics and CPG risk classification are summarized in Table 1. A total of 127 patients were included, with a median age of 66 years (range 47–78), and a median prostate-specific antigen of 7.7 (range 2.8–19.0). The CPG distribution from 1 to 3(GG2) was 20% (26/127), 57% (72/127), and 23% (29/127), respectively.

### Radiomics model performance

For the CPG-1+2+3(GG2) group, the radiomics model identified GP4Crib+ on a patient level with an area under the receiver operating characteristic curve (AUROC) of 0.68 (95% CI: 0.59‒0.77) and an area under the precision-recall curve (AUPRC) of 0.57 (95% CI: 0.46‒0.69) (GP4Crib+ prevalence of 0.41). For the CPG-2 and CPG-3(GG2) groups the model performed at an AUROC of 0.60 (95% CI: 0.46‒0.73) and 0.70 (95% CI: 0.45‒0.89), and a AUPRC of 0.57 (95% CI: 0.43‒0.74) (GP4Crib+ prevalence 0.46) and 0.68 (95% CI: 0.49‒0.91) (GP4Crib+ prevalence 0.55), respectively.

### Impact on patient selection for AS inclusion

The CPG-1+2, and the CPG-1+2+3(GG2) surveillance scenarios included, respectively, 98 and 127 patients. In these cohorts, GP4Crib+ was present in 27% (36/98) and 41% (52/127) of men, and 12% (12/98) and 17% (22/127) had a prostatectomy GG ≥ 3, respectively, making them unsuitable for AS under our assumption. In the CPG-1 group, where no radiomics model was applied, 88% (23/26) had no cribriform growth, and 100% (26/26) had no GG ≥ 3 at radical prostatectomy.

**Fig. 2:**
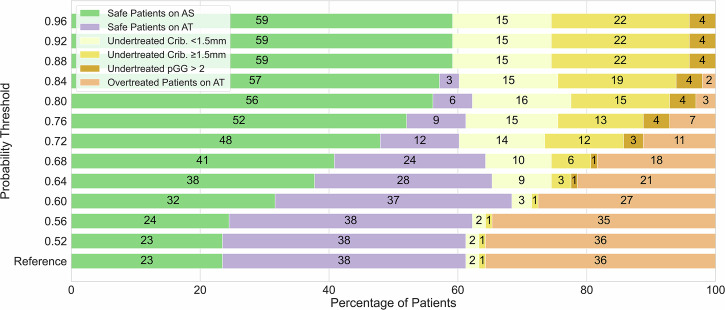
CPG-1+2 scenario: 98 CPG-1 and CPG-2 patients without radiomics-based evidence of cribriform growth for increasing model probability thresholds. The reference includes only CPG-1 patients selected into AS. Color palette, left to right: green = correctly in AS; purple =  correctly in AT; light yellow = falsely in AS (GP4Crib+ length < 1.5 mm); dark yellow = falsely in AS (GP4Crib+ length ≥ 1.5 mm); mustard =  falsely in AS (GP4Crib+ negative but prostatectomy GG ≥ 3); orange = falsely in AT. Numbers inside the bars represent probabilities. AS, Active surveillance; AT, Active treatment

For the CPG-1+2 scenario, 61% (60/98) of men would have been appropriately managed when excluding radiomics input, based on all CPG-1 men going to AS and all CPG-2 going to AT (reference). When applying the radiomics model to the CPG-2 patients in this group, a probability threshold of 0.60 yielded the most favorable balance between overtreatment (false positives) and undertreatment (false negatives) in comparison to above mentioned reference in our cohort (Fig. 2). At this threshold, the overtreatment (yellow bars) decreased substantially from 36% to 27% (absolute 9% difference) while the undertreatment (purple bars) increased from 3% to 4% (absolute 1% difference). This would have resulted in 69% of patients being appropriately managed compared to the 61% in the reference, while increasing the percentage eligible for AS from 26% to 36%. It is important to note that this threshold reflects cohort-specific optimization and is not proposed as a universal cutoff. The model’s probabilistic nature supports flexible threshold selection tailored to institutional protocols and clinical judgment. Increasing the radiomics model probability threshold for GP4Crib+ above 0.60 incrementally, decreased the overtreatment to 0% (false positives in orange), however, at the expense of completely losing appropriate active treatment guidance (true positives in purple) and increased undertreatment from 4% to 41% (false negatives in yellow). Notably, most undertreated cases at higher thresholds involved patients with small GP4Crib+ regions (maximum diameter of less than 1.5 mm). At a threshold of 0.64, the model failed to detect nearly three times as many patients with small cribriform areas compared to larger ones.

**Fig. 3:**
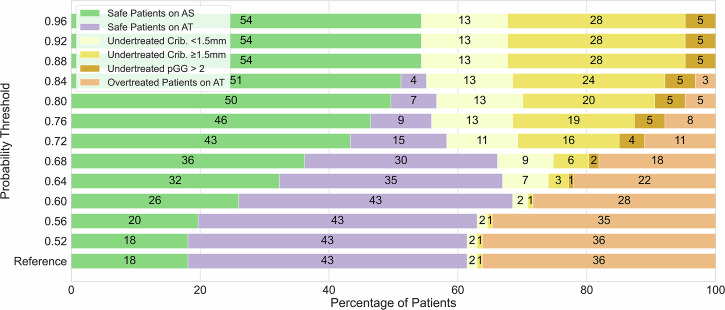
CPG-1+2+3(GG2): 127 CPG-1, CPG-2 and CPG-3 with GG2 without radiomics-based evidence of cribriform growth for increasing model probability thresholds. The reference represents only CPG-1 patients who are selected into AS. Color palette, left to right: green = correctly in AS; purple =  correctly in AT; light yellow = falsely in AS (GP4Crib+ length < 1.5 mm); dark yellow = falsely in AS (GP4Crib+ length ≥ 1.5 mm), mustard = falsely in AS (GP4Crib+ negative but prostatectomy GG ≥ 3); orange = falsely in AT. Numbers inside the bars represent percent probabilities. AS, Active surveillance; AT, Active treatment

For the CPG-1+2+3(GG2) scenario, 61% (78/127) of men would have been appropriately managed when excluding radiomics input (reference). When applying the radiomics model to the CPG-2 and CPG-3(GG2) patients in this group, a probability threshold of 0.60 yielded the most favorable balance between overtreatment (false positives) and undertreatment (false negatives) in comparison to the reference scenario in our cohort (Fig. 3). At this threshold, the overtreatment (yellow bars) decreased from 36% to 28% (absolute 8% difference) while the undertreatment (purple bars) remained at 3%. This would have resulted in 69% of patients being appropriately managed compared to the 61% in the reference, while increasing the percentage eligible for AS from 21% to 29%. Raising the radiomics probability threshold above 0.60 further decreased the overtreatment (false positives in orange) while rising undertreatment from a 3% to a 46% (false negatives in yellow). As in the previous scenario, most missed cases involved patients with small GP4Crib+ regions (maximum diameter smaller than 1.5 mm). At a threshold of 0.64, the model failed to detect slightly more than twice as many patients with small cribriform regions (from 2% to 7%) compared to those with larger cribriform regions present (from 1% to 3%). This higher threshold also increased the number of patients safely allocated to AS (32%, green color) compared to the reference scenario (18%, only CPG-1 sent to AS), while also reducing overtreatment from 36% to 22% (absolute 14% difference).

## Discussion

The aim of this study was to evaluate the clinical potential of an MRI-based radiomics model—previously trained to identify cribriform growth at a GP level—when applied to the entire prostate gland, in order to simulate its impact on patient-level AS eligibility decisions under hypothetical treatment scenarios. By generating voxel-wise GP4Crib+ probability maps and aggregating them across the prostate, we evaluated whether this approach could exclude GP4Crib+ patients from AS in two hypothetical scenarios assuming that CPG-2 or CPG-3(GG2) patients without cribriform growth could be managed with AS: one including CPG-1 and selected CPG-2 patients, and another further including selected CPG-3 patients with GG 2 (CPG-1+2+3(GG2)). In both, inclusion depended on the absence of radiomics-predicted cribriform growth, with the CPG-1 group serving as a fixed reference cohort assigned to AS by default.

The CPG-1+2 scenario aligns with current practice in several institutions that consider selected CPG-2 patients for AS, typically following multidisciplinary review and favorable imaging features [12, 13]. The CPG-1+2+3(GG2) scenario explores a more stretched AS eligibility, expanding to include CPG-3 patients with GG2, dependent on the absence of radiomics-predicted cribriform growth. Importantly, for both scenarios, the radiomics model was only applied to CPG-2 and CPG-3(GG2) patients. The CPG-1 cohort served as a fixed reference group of patients, applicable to AS in current practice. This setup allowed for assessment of how radiomics-informed exclusion of cribriform growth could affect AS eligibility under hypothetical assumptions, rather than propose expansion of AS itself.

Previous research has shown associations between cribriform growth and MRI-derived features but has largely remained observational or diagnostic in nature [3, 16]. These efforts did not explore the integration of radiomics predictions into treatment decision-making frameworks. In contrast, our study applies an independently developed radiomics model to simulate patient-level treatment decisions.

While the radiomics model achieved moderate performance in detecting GP4Crib+ on a patient level, our results show that its application within these structured clinical scenarios yielded clear net benefits. Specifically, at a probability threshold of 0.60, the CPG-1+2 scenario reduced overtreatment by 9% with only a 1% increase in undertreatment compared to the benchmark scenario of referring only CPG-1 patients to AS. Similarly, the CPG-1+2+3(GG2) scenario achieved an 8% net benefit, maintaining undertreatment at the same 3% level as the reference. These findings demonstrate that even with limited standalone predictive performance, a radiomics model can meaningfully inform treatment decisions when embedded within structured eligibility frameworks.

Importantly, this study does not propose a universal or fixed threshold for GP4Crib+ classification. Instead, the continuous probability output allows clinicians to tailor decision thresholds according to local standards (*i.e.*, institutional protocols, physician judgment and patient preference). Institutions with robust AS follow-up protocols might accept higher thresholds—thus minimizing overtreatment—while others may prioritize minimizing undertreatment by applying more conservative cutoffs. This tradeoff emphasizes the utility of a model that does not dictate a binary decision but rather offers an interpretable risk estimate that can support individualized care pathways. Nevertheless, formal decision-curve analysis may complement this approach and could be valuable in future prospective studies, particularly when standardized thresholds or patient-level risk models are established.

An additional consideration is the group of patients who were GP4Crib- (both by model and histology) but had GG ≥ 3 at prostatectomy. These patients were appropriately predicted as negative by the model, but would still be ineligible for AS under our criteria and thus considered undertreated. This illustrates the limitation that our radiomics model was previously developed specifically to identify the presence of GP4Crib+ based on ADC features and was not trained to detect other adverse histologic features, such as GG ≥ 3. Therefore, the model’s predictions were used only to evaluate the presence or absence of GP4Crib+, while decisions regarding the appropriateness of AS also accounted for final prostatectomy GG, based on the assumption that patients without cribriform growth and with GG < 3 may be suitable candidates for AS under close monitoring. Future work may explore expanding radiomics models to address multiple adverse features simultaneously.

It’s also noteworthy that many of the model’s false negatives—patients incorrectly predicted as GP4Crib- —had very small cribriform foci (< 1.5 mm), which may be below the detection limit of MRI. The prognostic significance of such small cribriform lesions remains uncertain, and further studies are required to clarify whether missing them impacts long-term outcomes. Until more is known, cautious interpretation of negative predictions in the presence of GP4Crib+ in low volumes is warranted. Finally, while our results are promising, they stem from a retrospective, single-center study and should be validated in larger, prospective, and multicenter cohorts to ensure generalizability.

Although most MRIs met PI-RADS v2.1. acquisition standards, a small number of cases exhibited minor deviations in slice thickness, in-plane resolution, or timing parameters, reflecting protocol variability over the 10-year acquisition period. Additionally, while the majority of scans were acquired at 3 T, a limited number were performed at 1.5 T. Despite this heterogeneity in acquisition parameters and field strength, the model performed robustly. Nevertheless, standardized imaging protocols (*e.g.*, choice of *b*-values) may improve reproducibility and should be prioritized in future prospective validation studies.

In conclusion, this study serves as a proof-of-concept, demonstrating that an MRI-based radiomics model for detecting cribriform growth can inform hypothetical AS eligibility scenarios in men with intermediate-risk prostate cancer. Under the assumption that CPG-2 and CPG-3(GG2) patients without cribriform growth can be safely managed with AS, the model’s integration into structured decision scenarios showed a potential to reduce overtreatment without increasing undertreatment. While the findings are preliminary and based on retrospective single-center data, they provide early evidence supporting the model’s utility in risk-adapted prostate cancer care. The model’s probabilistic output represents a potentially flexible and interpretable biomarker, with promise to support individualized decision-making once validated in real-world settings.

## Data Availability

Data that support the findings of this study are not openly available due to reasons of sensitivity.

## Abbreviations

AS: Active surveillance
ADC: Apparent diffusion coefficient
AUROC: Area under the receiver operating characteristic curve
CPG: Cambridge Prognostic Group
CI: Confidence intervals
GP4Crib+: Cribriform growth
DWI: Diffusion-weighted imaging
GG: Gleason grade
GP: Gleason pattern

## Footnotes

1 https://github.com/OSIPI/TF2.4_IVIM-MRI_CodeCollection/tree/main/src/original/PvH_KB_NKI

## References

[1] Padhani AR, Schoots IG (2023) Imaging-based diagnostic and therapeutic strategies for prostate cancer in the coming decades. Radiology 307: e222990. 10.1148/radiol.222990 37249432 10.1148/radiol.222990

[2] Schoots IG, Padhani AR (2020) Delivering clinical impacts of the MRI diagnostic pathway in prostate cancer diagnosis. Abdom Radiol (NY) 45:4012–4022. 10.1007/s00261-020-02547-x 32356003 10.1007/s00261-020-02547-x PMC7716818

[3] Seyrek N, Hollemans E, Schoots IG, Van Leenders GJLH (2023) Association of quantifiable prostate MRI parameters with any and large cribriform pattern in prostate cancer patients undergoing radical prostatectomy. Eur J Radiol 166:110966. 10.1016/j.ejrad.2023.110966 37453276 10.1016/j.ejrad.2023.110966

[4] Cai Q, Costa DN, Metter CK et al (2022) Sensitivity of multiparametric MRI and targeted biopsy for detection of adverse pathologies (Cribriform Gleason pattern 4 and intraductal carcinoma): correlation of detected and missed prostate cancer foci with whole mount histopathology. Urol Oncol 40:452.e451–452.e458. 10.1016/j.urolonc.2022.07.012 10.1016/j.urolonc.2022.07.012 36008255

[5] Tonttila PP, Ahtikoski A, Kuisma M, Pääkkö E, Hirvikoski P, Vaarala MH (2019) Multiparametric MRI prior to radical prostatectomy identifies intraductal and cribriform growth patterns in prostate cancer. BJU Int 124:992–998. 10.1111/bju.14812 31102571 10.1111/bju.14812

[6] Chen Z, Pham H, Abreu A et al (2021) Prognostic value of cribriform size, percentage, and intraductal carcinoma in Gleason score 7 prostate cancer with cribriform Gleason pattern 4. Hum Pathol 118:18–29. 10.1016/j.humpath.2021.09.005 34543668 10.1016/j.humpath.2021.09.005

[7] Hollemans E, Verhoef EI, Bangma CH et al (2019) Large cribriform growth pattern identifies ISUP grade 2 prostate cancer at high risk for recurrence and metastasis. Mod Pathol 32:139–146. 10.1038/s41379-018-0157-9 30349027 10.1038/s41379-018-0157-9 PMC6300553

[8] Kweldam CF, Wildhagen MF, Steyerberg EW, Bangma CH, Van Der Kwast TH, Van Leenders GJ (2015) Cribriform growth is highly predictive for postoperative metastasis and disease-specific death in Gleason score 7 prostate cancer. Mod Pathol 28:457–464. 10.1038/modpathol.2014.116 25189638 10.1038/modpathol.2014.116

[9] Iczkowski KA, Torkko KC, Kotnis GR et al (2011) Digital quantification of five high-grade prostate cancer patterns, including the cribriform pattern, and their association with adverse outcome. Am J Clin Pathol 136:98–107. 10.1309/ajcpz7wbu9yxsjpe 21685037 10.1309/AJCPZ7WBU9YXSJPE PMC4656017

[10] Van Leenders GJLH, Kweldam CF, Hollemans E et al (2020) Improved prostate cancer biopsy grading by incorporation of invasive cribriform and intraductal carcinoma in the 2014 grade groups. Eur Urol 77:191–198. 10.1016/j.eururo.2019.07.051 31439369 10.1016/j.eururo.2019.07.051

[11] Network NCC (2023) Prostate Cancer (Version 3.2023). Available via https://www.nccn.org/guidelines/guidelines-detail?category=1&id=1459

[12] European Association of Urology (2025) EAU Guidelines: Prostate Cancer. EAU Guidelines Office, Arnhem, The Netherlands. Available via https://uroweb.org/guidelines/prostate-cancer

[13] National Institute for Health and Care Excellence (2019) Prostate cancer: diagnosis and management (2019). Available via https://www.nice.org.uk/guidance/ng131/resources/prostate-cancer-diagnosis-and-management-pdf-66141714312133 31393679

[14] Bernardino RM, Sayyid RK, Lajkosz K et al (2024) Limitations of prostate biopsy in detection of cribriform and intraductal prostate cancer. Eur Urol Focus 10:146–153. 10.1016/j.euf.2023.08.010 37696743 10.1016/j.euf.2023.08.010

[15] Ericson KJ, Wu SS, Lundy SD, Thomas LJ, Klein EA, McKenney JK (2020) Diagnostic accuracy of prostate biopsy for detecting cribriform Gleason pattern 4 carcinoma and intraductal carcinoma in paired radical prostatectomy specimens: implications for active surveillance. J Urol 203:311–319. 10.1097/ju.0000000000000526 31483693 10.1097/JU.0000000000000526

[16] Fernandez Salamanca M, Simões R, Deręgowska-Cylke M et al (2025) Beyond Gleason grading: MRI radiomics to differentiate cribriform growth from non-cribriform growth in prostate cancer men. MAGMA 38:817–827. 10.1007/s10334-025-01251-5 40299156 10.1007/s10334-025-01251-5 PMC12497665

[17] Kooreman ES, Van Houdt PJ, Keesman R et al (2021) Daily intravoxel incoherent motion (IVIM) in prostate cancer patients during MR-guided radiotherapy—a multicenter study. Front Oncol 11:705964. 10.3389/fonc.2021.705964 34485138 10.3389/fonc.2021.705964 PMC8415108

[18] Isensee F, Jaeger PF, Kohl SAA, Petersen J, Maier-Hein KH (2021) nnU-Net: a self-configuring method for deep learning-based biomedical image segmentation. Nat Methods 18:203–211. 10.1038/s41592-020-01008-z 33288961 10.1038/s41592-020-01008-z

[19] The Cancer Imaging Archive (2020) Prostate MRI and ultrasound with pathology and coordinates of tracked biopsy (Prostate-MRI-US-Biopsy) (version 2)[Data set]. 10.7937/TCIA.2020.A61IOC1A

